# Differential sensitivity of epidermal cell subpopulations to β-catenin**-**induced ectopic hair follicle formation

**DOI:** 10.1016/j.ydbio.2010.04.005

**Published:** 2010-07-01

**Authors:** Christopher M. Baker, Annemieke Verstuyf, Kim B. Jensen, Fiona M. Watt

**Affiliations:** a CRUK Cambridge Research Institute, Li Ka Shing Centre, Robinson Way, Cambridge CB2 0RE, UK; b Laboratory of Experimental Medicine and Endocrinology, KULeuven, Herestraat 49 bus 902, 3000 Leuven, Belgium; c Wellcome Trust Centre for Stem Cell Research, Tennis Court Road, Cambridge CB2 1QR, UK

**Keywords:** Wnt, Vitamin D receptor, Stem cells, Differentiation, Epidermis, Niche

## Abstract

Wnt signalling is required for hair follicle development and for the growth phase (anagen) of postnatal follicles. When the pathway is activated at high levels in adult mouse epidermis, ectopic follicles form from existing follicles, interfollicular epidermis (IFE) and sebaceous glands, revealing a remarkable ability of the tissue to be reprogrammed. To compare the competence of different epidermal cell populations to form ectopic follicles, we expressed a 4-hydroxy-tamoxifen (4OHT) inducible, stabilised β-catenin transgene (ΔNβ-cateninER) under the control of two different promoters. We targeted the reservoir of stem cells in the hair follicle bulge via the keratin 15 (K15) promoter and targeted the sebaceous glands and base of the follicle (bulb) with a truncated K5 promoter (ΔK5). No ectopic follicles formed in the IFE in either model, establishing the autonomy of the IFE stem cell compartment in undamaged epidermis. Activation of β-catenin in the bulge stimulated proliferation and bulge expansion. Existing hair follicles entered anagen, but no ectopic follicles formed. ΔK5ΔNβ-cateninER expressing hair follicles also entered anagen on 4OHT treatment. In addition, a subpopulation of cells at the base of the sebaceous gland readily formed ectopic follicles, resulting in complete and reversible conversion of sebaceous glands into hair follicles. Combined activation of β-catenin and the vitamin D receptor enhanced differentiation of sebaceous gland-derived hair follicles and stimulated ectopic follicle formation in the hair follicle bulb, but not in the bulge. Our results suggest that the bulge and sebaceous gland are, respectively, non-permissive and permissive niches for Wnt induced hair follicle differentiation.

## Introduction

Adult mammalian epidermis is maintained by several distinct populations of stem cells. They occupy different locations, including the hair follicle bulge, sebaceous gland, and the junctional zone that lies between the sebaceous glands, interfollicular epidermis and bulge (Owens and Watt, 2003; Watt and Jensen, 2009; Fuchs, 2009). Under normal homeostatic conditions, each stem cell population maintains only the differentiated lineages appropriate for its location. However, in response to injury or genetic modification, stem cells in any location are capable of giving rise to progeny that differentiate along all epidermal lineages (Watt and Jensen, 2009).

One of the key pathways that regulates epidermal lineage selection is the canonical Wnt pathway (Millar, 2002; Fuchs and Nowak, 2008). Wnt signalling is required for hair follicle development and for the growth phase (anagen) of postnatal follicles. When the pathway is inhibited, hair follicles are no longer maintained, but convert into cysts of IFE with associated sebocytes (Huelsken et al., 2001; Merrill et al., 2001; Andl et al., 2002; Niemann et al., 2002). Conversely, high levels of canonical Wnt activity can induce formation of ectopic hair follicles. When stabilised, N-terminally truncated β-catenin (ΔNβ-catenin) is expressed in the epidermis under the control of the K14 promoter, ectopic follicles form in homozygous but not in heterozygous animals (Gat et al., 1998; Lowry et al., 2005).

Ectopic hair follicle formation reflects reprogramming of adult stem cell progeny to select the hair follicle lineages and indicates a remarkable plasticity of adult epidermis (Silva-Vargas et al., 2005; Ito et al., 2007; Watt and Collins, 2008). One strategy to examine this process is to express a 4-hydroxy-tamoxifen (4OHT) inducible β-catenin transgene (ΔNβ-cateninER) under the control of the K14 promoter (Van Mater et al., 2003; Lo Celso et al., 2004). β-catenin is selectively activated in adult epidermis by topical application of 4-hydroxy-tamoxifen (4OHT), and different doses of 4OHT can be used to achieve different levels of β-catenin activation (Silva-Vargas et al., 2005).

The keratin 14 promoter is active in the basal layer of the interfollicular epidermis (IFE), the hair follicle outer root sheath (ORS) and at the periphery of the sebaceous gland (SG) (Gat et al., 1998; Lo Celso et al., 2004). Therefore studies of K14ΔNβ-catenin or K14ΔNβ-cateninER transgenic mice do not reveal how activation of the canonical Wnt pathway in one group of cells impacts on ectopic follicle formation in other regions. Although bulge stem cells contribute to the normal hair growth cycle it is unknown whether they are also required for ectopic hair follicle formation (Ito et al., 2005). Lineage tracing has established that ectopic follicles in the IFE can arise from IFE cells (Silva-Vargas et al., 2005), but it is unclear whether some IFE follicles are HF derived. Finally, SG gland-derived ectopic HF could potentially arise from stem cells in the bulge (Horsley et al., 2006) or in the junctional zone (Jensen et al., 2009). In order to investigate these issues, we have expressed the ΔNβ-cateninER transgene under the control of two different promoters. We used the K15 promoter to target bulge stem cells (Liu et al., 2003) and a truncated K5 (ΔK5) promoter to target the HF ORS and SG (Brown et al., 1998). Unexpectedly, ΔK5 promoter activity was not detected in the bulge; the construct was primarily expressed in the SG and HF bulb.

## Materials and methods

### Transgene construction

The 1320 bp ∆K5 promoter, generously provided by A. Balmain, UCSF, San Francisco, USA (Brown et al., 1998) was amplified using Roche Pwo Superyield DNA Polymerase, with primers containing AvaI overhangs (5′ CCG GAC TGG GAG CTG GGC TGA G and 3′ TCG GGG TGC TGG AGA GAA AGA GC). The PCR product was then cleaved using AvaI. The K14 promoter was excised from an expression cassette kindly provided by E. Fuchs, The Rockefeller University, New York, USA (Vasioukhin et al., 1999) using AvaI, its ends dephosphorylated with calf intestine phosphatase, and the ∆K5 promoter ligated in its place.

The 4968 bp K15 promoter, generously provided by G. Cotsarelis, University of Pennsylvania, Philadelphia, USA (Liu et al., 2003), was excised from its expression vector using XmaI and EcoRI, and blunt ended with mung bean nuclease. The K14 expression cassette, from which the K14 promoter had been excised, was also blunt ended and dephosphorylated, before blunt end ligation with the K15 promoter.

To generate the transgene construct, ∆Nβ-catenin and ER were amplified separately from the T2ER pBabepuro retroviral vector (Lo Celso et al., 2004) using the following primers: 5′CGG GAT CCA TGG CAA TCC CCG AGC TGA C and 3′ TAG CTA GCC GCA AGT CAG TGT CAA ACC (∆Nβ-catenin), 5′ATG CTA GCA CGA AAT GAA ATG AAA TGG GTG and 3′CGG GAT CCT CAG ATC GTG TTG GGG AAG C (ER). This resulted in creation of BamHI sites at the 5′ end of ∆Nβ-catenin and the 3′ end of ER and Nhe1 sites at the 3′ end of ∆Nβ-catenin and 5′ end of ER. The PCR products were digested with BamH1 and Nhe1 and ligated into the K15 and ∆K5 expression cassettes.

A HindIII site in ∆Nβ-catenin was removed using the Stratagene Quikchange XL site-directed mutagenesis kit and primers 5′CTG GTG GAA TGC AAG CGT TAG GAC TCC ATC TCA C and 3′GTG AGA TGG AGT CCT AAC GCT TGC ATT CCA CCA G. Each transgene construct was excised as an EcoRI/HindIII fragment, gel purified (Qiagen), further purified by phenol:chloroform extraction, and then resuspended at a concentration of 5 µg/ml in sterile injection buffer (10 mM Tris–HCl, 0.1 mM EDTA, pH 7.4) for pronuclear injection.

### Generation of transgenic mice and determination of copy number

Each transgene construct was injected into the male pronucleus of day-1 fertilised (CBA × C57BL/6) F1 embryos. Animals were screened for the presence of transgene by PCR of ear biopsy DNA with primers specific for β-catenin (ATG CTG CTG GCT GGC TAT GGT CAG) and ER (GCA CAC AAA CTC TTC ACC). Founder lines were tested for germline transmission of each transgene. Transgene copy numbers were determined using Applied Biosystems 7900HT Fast Real Time PCR (Ballester et al., 2004).

Data presented are from ΔK5∆Nβ-cateninER line 6470/1.3F (46 copies) and K15∆Nβ-cateninER line 6512.4 (6 copies). ΔK5∆Nβ-cateninER lines 6577A.2 (2 copies) and 6470/1.20M (8 copies) had a milder phenotype than line 6470/1.3F. K15∆Nβ-cateninER.6512.2 (6 copies) had a similar phenotype to line 6512.4.

### Experimental procedures on mice

All experiments were subject to CR-UK ethical review and performed under the terms of a UK Government Home Office licence. At the start of each experiment mice were 8 weeks old, and therefore in the resting phase of the hair cycle (Stenn and Paus, 2001). The ∆Nβ-cateninER transgene was activated by topical application of 4-hydroxy-tamoxifen (4OHT; Sigma). 4OHT was dissolved in acetone at a concentration of 15 mg/ml. 100 μl was applied per mouse to the tail and 100 μl to clipped dorsal skin every other day. 4OHT treated wild type littermates and acetone treated transgenics were used as controls. No sex-specific effects of 4OHT were observed.

In some experiments, mice were treated topically with 200 ng (in 100 μl acetone) of the 17-methyl D-ring-1,25-dihydroxyvitamin D3 analogue CD578, 30 min prior to 4OHT treatment. CD578 was a kind gift from P. DeClerq (University of Gent, Belgium) (Wu et al., 2002).

### Primary antibodies

Antibodies against the following antigens were used: CCAAT displacement protein (CDP, Santa Cruz Biotechnology), fatty acid synthase (IBL), keratin 14 (MK14; rabbit; Covance), keratin 14 (LL002; mouse; Abcam), Lrig1 (goat polyclonal; R & D Systems), keratin 15 (LHK15; mouse; kind gift of I.M. Leigh), keratin 31 (Progen Biotechnik), Ki67 (Thermo Scientific), Lef1 (Upstate), Jagged 1 (Santa Cruz Biotechnology) and S100A3 (Stratech Scientific Limited).

### Histochemistry and immunohistochemistry

Tail epidermal whole mounts were prepared as described previously (Braun et al., 2003). Tissue samples for sectioning were either fixed overnight in neutral buffered formalin and embedded in paraffin wax, or, alternatively, frozen, unfixed, in OCT compound (Raymond A. Lamb, UK) on a frozen isopentane surface (cooled with dry ice). 5 µm sections were prepared and stained with Haematoxylin and Eosin.

Immunohistochemistry on paraffin wax sections was performed as described previously (Niemann et al., 2002). Briefly, antigen retrieval was performed by boiling in 10 mM sodium citrate buffer (pH 6) for 20 min and non-specific binding was blocked by incubating the sections in 10% bovine serum and 3% fetal calf serum for 1 h. Primary antibodies were diluted in blocking mix, incubated overnight at 4 °C and detected using fluorescently conjugated secondary antibodies (Invitrogen).

For immunostaining frozen sections with all antibodies, the sections were air dried for 5 min, then fixed in 4% paraformaldehyde (Sigma) for 10 min, permeabilised with 0.1% Triton X-100 (Sigma) for 5 min, blocked for 60 min in 2% BSA (Sigma), 1% milk powder and 0.25% fish skin gelatin (FSG), then incubated overnight with primary antibodies.

Ki67 staining was carried out on Leica Bondmax Immunostainer. Endogenous alkaline phosphatase activity was visualised in frozen sections using the Sigma Alkaline Phosphatase Leukocyte kit, with FRV-alkaline solution (red), and counterstained with haemotoxylin.

All fluorescent sections were analysed on a Leica TCS SP5 confocal microscope. All H&E and alkaline phosphatase stained sections were scanned using a Scanscope XT (Aperio).

### In situ hybridisation

In situ hybridisation was performed as described previously (Poulsom et al., 1998), using ^35^S-labeled riboprobes to ∆Nβ-cateninER. The ∆Nβ-cateninER probe was generated by digesting the transgene construct with Pst1, and inserting the resultant 760 bp DNA fragment into pgem3z vector. Hybridisation with a β-actin antisense probe served as a positive control. In situ images were photographed with an Olympus BX51 microscope and colour switched to red in Adobe Photoshop to distinguish the positive signal from black pigmentation in melanocytes.

## Results

### The K15 and ΔK5 promoters target the ΔNβ-cateninER transgene to different epidermal locations

**Fig. 1:**
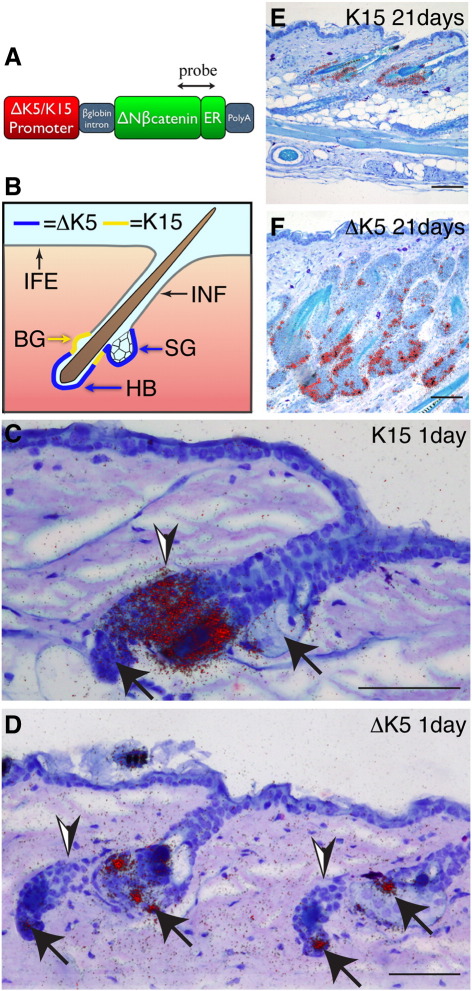
Sites of transgene expression. (A) Schematic of the ΔK5 and K15ΔNβ-cateninER constructs. Double headed arrow indicates region detected with the in situ hybridisation probe. (B) Schematic showing sites of promoter activity. The ΔK5 promoter targets the hair follicle bulb (HB) and sebaceous glands (SG) but not the bulge (BG). The K15 promoter targets the hair follicle bulge. Neither promoter is active in the interfollicular epidermis (IFE) or infundibulum (INF). (C–F) In situ hybridisation of back skin sections of K15ΔNβ-cateninER (K15) and ∆K5ΔNβ-cateninER (ΔK5) transgenic mouse lines treated with 4OHT for (C, D) 1 day or (E, F) 21 days. Arrowheads indicate position of bulge; arrows mark hair bulb and sebaceous glands. Silver grains in darkfield images are false coloured red, superimposed on haemotoxylin staining. Scale bars: 50 µm.

The transgene cassettes used in this study and their reported sites of expression are shown schematically in Figs. 1A, B. Multiple independent transgenic lines were generated for each construct and gave similar phenotypes, thereby ruling out effects of integration site. The sites of transgene expression were confirmed by radioactive in situ hybridisation using a probe spanning the boundary between the β-catenin C-terminus and the estrogen receptor (ER) (Figs. 1A, C–F) and by immunolabelling the epidermis with antibodies to the ER epitope (Silva-Vargas et al., 2005; data not shown).

As expected from previous studies (Liu et al., 2003), the K15 promoter was only active in the HF bulge, a well-characterized reservoir of quiescent stem cells that express K15, CD34 and a range of other markers (Figs. 1B, C, E; Trempus et al., 2003; Tumbar et al., 2004; Cotsarelis, 2006; Watt and Jensen, 2009). Promoter activity was restricted to the bulge, both in untreated transgenic mice and when the skin was treated with 4OHT to activate the β-catenin transgene (Figs. 1C, E and data not shown).

In adult mice, the ΔK5 promoter is known to be active almost exclusively in the hair follicle (Brown et al., 1998). Consistent with this, we did not observe expression in the IFE or infundibulum, which connects the hair follicle with the IFE. ΔK5ΔNβ-cateninER was expressed at the periphery of the SG, along the ORS and in the hair follicle bulb (Figs. 1B, D). We did not detect expression in the bulge, even though bulge stem cells express keratin 5 (Fig. 1D, arrowheads). Following 4OHT treatment the transgene continued to be expressed in existing and ectopic hair follicles but not in the IFE (Fig. 1F).

### β-catenin activation induces expression of known Wnt target genes

**Fig. 2:**
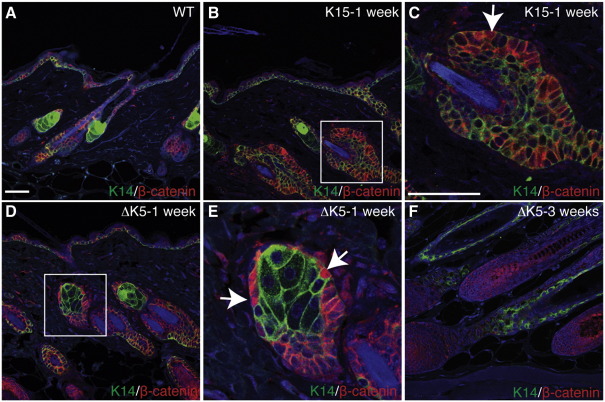
Location of cells with nuclear β-catenin. Sections of wild type telogen back skin (A) or transgenic back skin that had received repeated 4OHT applications for the number of weeks shown (B–F) were labeled with antibodies to keratin 14 (green) and β-catenin (red) and counterstained with DAPI (blue). Boxed regions in B, D are shown at higher magnification in C, E, respectively. Arrows indicate β-catenin positive nuclei. (F) Nuclear β-catenin in cells at the base of an anagen follicle. Scale bar: 100 µm.

In order to confirm that β-catenin signalling was active in both transgenic models, back skin was treated for up to 21 days with 4OHT and then stained with antibodies to β-catenin (Fig. 2). In wild type epidermis, most β-catenin was localized at cell–cell borders; the only site of active canonical Wnt signalling, as indicated by nuclear β-catenin, was in the bulb of anagen follicles (Gat et al., 1998; Lo Celso et al., 2004; Figs. 2A, F and data not shown). In 4OHT treated K15ΔNβ-cateninER epidermis nuclear β-catenin was detected in cells in the bulge (Figs. 2B, C), whereas in ΔK5ΔNβ-cateninER epidermis the most prominent site of nuclear β-catenin was in cells at the periphery of the SG (Figs. 2D–E).

**Fig. 3:**
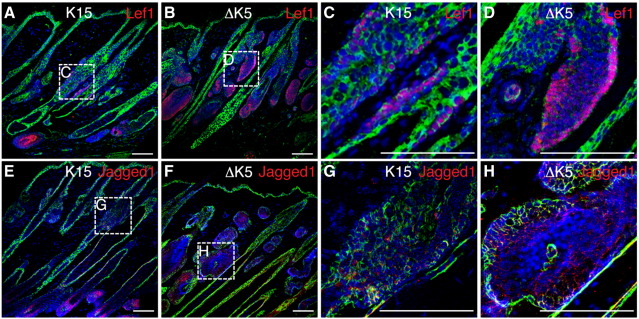
Expression of β-catenin target genes Lef1 and Jagged1. Immunofluorescence labeling of sections of K15ΔNβ-cateninER (K15) and ∆K5ΔNβ-cateninER (ΔK5) back skin treated with 4OHT for 21 days. Antibodies detected (A–D) Lef1 (red), (E–H) Jagged1 (red). Sections were also labeled with anti-keratin 14 (green) and counterstained with DAPI (blue) to visualise nuclei. Boxed regions in A, B, E, F are shown at higher magnification in C, D, G, H, respectively. Scale bars: 50 µm.

As further confirmation of the sites of Wnt signalling activity, back skin was treated for 21 days with 4OHT and then labeled with antibodies to Lef1 and Jagged1, two well-characterized β-catenin target genes (Filali et al., 2002; Estrach et al., 2006) (Fig. 3). As reported previously (Huelsken et al., 2001; Merrill et al., 2001; Niemann et al., 2002; Jamora et al., 2003; Estrach et al., 2006; Nowak et al., 2008), in wild type skin Lef1 and Jagged1 were expressed most abundantly in the bulb of anagen follicles, where endogenous Wnt signalling is most active (data not shown). In K15ΔNβ-cateninER transgenic skin there was upregulation of Lef1 (Figs. 3A, C) and Jagged1 (Figs. 3E, G) in the bulge. In ΔK5ΔNβ-cateninER transgenic skin Jagged1 and Lef1 were upregulated in the sebaceous gland and HF bulb (Figs. 3B, D, F, H). We conclude that targets of the canonical Wnt pathway are activated in those cells that express the β-catenin transgene under the control of the K15 or ΔK5 promoter.

### Different consequences of activating β-catenin in the bulge, sebaceous gland and outer root sheath

**Fig. 4:**
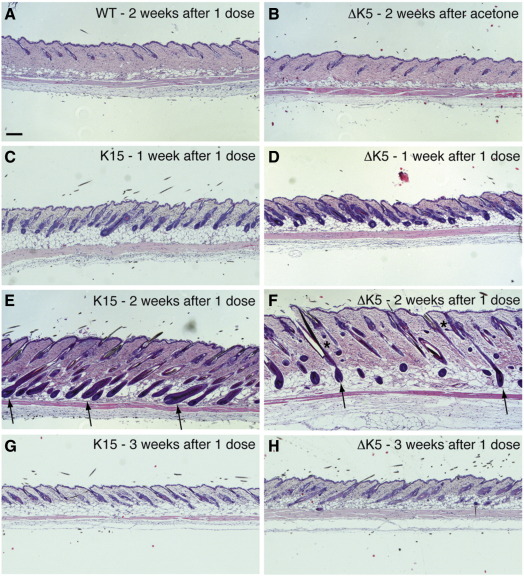
Effects of a single dose of 4OHT. Haematoxylin and eosin stained sections of back skin from telogen mice that had been treated once with acetone (B) or 4OHT (A, C–H) and collected 1–3 weeks later. WT: wild type. Arrows show anagen follicles. Asterisks show ectopic follicles. Scale bar: 100 µm.

A single application of 4OHT is sufficient to drive hair follicles into anagen in K14ΔNβ-cateninER transgenic mice (Van Mater et al., 2003; Lo Celso et al., 2004). However, the specific cells that are involved have not been defined. To examine this, we treated transgenic mice in the resting (telogen) phase of the hair cycle with a single dose of 4OHT and monitored the effects on the epidermis for up to three weeks (Fig. 4). 4OHT treated wild type mice and transgenic mice treated with vehicle (acetone) alone did not enter anagen during this period (Figs. 4A, B and data not shown). We observed that hair follicles in both K15ΔNβ-cateninER and ΔK5ΔNβ-cateninER epidermis had begun to enter anagen 1 week after 4OHT treatment (Figs. 4C, D), and by 2 weeks the majority of follicles were in full anagen (arrows, Figs. 4E, F). At 2 weeks ectopic follicles arising from the SG were observed in ΔK5ΔNβ-cateninER mice (Fig. 4F, asterisks); however, no ectopic follicles were observed in K15ΔNβ-cateninER mice (Fig. 4E). By 3 weeks after 4OHT treatment both K15ΔNβ-cateninER and ΔK5ΔNβ-cateninER epidermis had re-entered telogen. We conclude that expression of β-catenin under the control of either the K15 or the ΔK5 promoter is sufficient to drive anagen, indicating that there is no absolute requirement for direct activation of bulge stem cells.

**Fig. 5:**
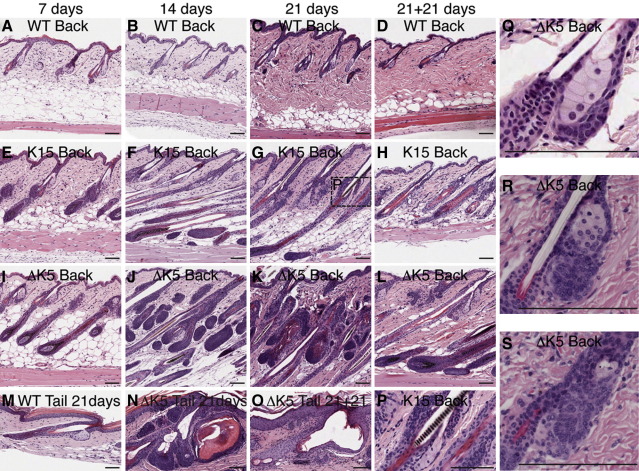
Effects of repeated doses of 4OHT. Haematoxylin and eosin stained sections of wild type (WT), K15ΔNβ-cateninER (K15) and ∆K5ΔNβ-cateninER (ΔK5) transgenic skin treated with 4OHT for 7 days (A, E, I, Q, R), 14 days (B, F, J, S), 21 days (C, G, K, M, N, P), or 21 days + 21 days recovery (D, H, L, O). Note cysts in (N, O) and conversion of sebaceous glands into ectopic hair follicles in (Q–S). Scale bars: 50 µm.

In order to examine ectopic follicle formation in more detail we next investigated the effects of repeated applications of 4OHT. Back skin was treated for up to three weeks, starting when the mice were 8 weeks old and in the telogen phase of the hair growth cycle (Figs. 5A–L). Wild type mice remained in telogen throughout the 4OHT treatment period and for three weeks after treatment had ceased (Figs. 5A–D). In contrast, K15ΔNβ-cateninER and ΔK5ΔNβ-cateninER transgenic mouse skin entered anagen within 7 days of treatment (Figs. 5E, I). When the skin was analysed 21 days after 4OHT treatment had ended, K15ΔNβ-cateninER follicles were exiting anagen (Fig. 5H), while anagen persisted in ΔK5ΔNβ-cateninER skin (Fig. 5L).

In K15ΔNβ-cateninER mice no ectopic follicles formed, the only effect of β-catenin activation, in addition to anagen induction, being swelling of the bulge from 14 days onwards (Figs. 5F, G, P). Once 4OHT treatment stopped the bulge returned to its normal size (Fig. 5H). In contrast, ectopic follicles were readily visible from 14 days in ΔK5ΔNβ-cateninER mice and became progressively enlarged and more mature by 21 days (Figs. 5J, K). As in the case of K14ΔNβ-cateninER mice (Lo Celso et al., 2004), ectopic follicles regressed partially but not completely when 4OHT treatment was stopped (Fig. 5L).

In tail skin, as in back, anagen was induced by activation of either transgene (Figs. 5M, N; data not shown). In K15ΔNβ-cateninER transgenic epidermis swelling of the bulge was observed (data not shown). However, as in back skin, ectopic follicles only developed when the transgene was expressed under the control of the ΔK5 promoter (Fig. 5N). In tail skin some ΔK5ΔNβ-cateninER transgenic mice developed large cysts, which were not observed in back skin and persisted after 4OHT treatment had stopped (Figs. 5N, O).

In ΔK5ΔNβ-cateninER mice, ectopic follicles formed from the SG and ORS but not from the IFE or bulge (Figs. 5J, K). To quantitate ectopic hair follicle formation, sections of ΔK5ΔNβ-cateninER skin that had been treated with 4OHT for 21 days were immunolabelled with antibodies to CDP, which is expressed in ectopic follicles (Lo Celso et al., 2004; Silva-Vargas et al., 2005). For 100 pre-existing HF, examined in sections of back skin from three different mice, we scored the number of CDP positive outgrowths from SG, ORS and hair follicle bulb (HB), all sites in which the promoter is active (Figs. 1–3). No ectopic follicles were detected emanating from the bulb (Table 1). 5% of ectopic follicles arose from the ORS, but the overwhelming majority of ectopic follicles arose from the SG (Table 1). Within the SG, ectopic follicles arose from a small group of undifferentiated cells in the lower SG that lacked expression of the junctional zone stem cell marker Lrig1 (Figs. 5Q–S and data not shown; Jensen et al., 2009).

**Fig. 6:**
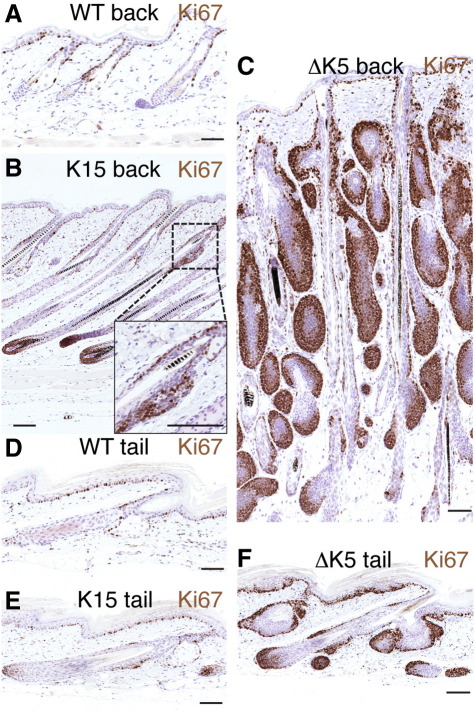
Activation of β-catenin stimulates proliferation in transgene positive epidermal cells. Immunohistochemical labeling with anti-Ki67 (brown) of back and tail skin from wild type (WT) (A, D), K15ΔNβ-cateninER (K15) (B, E) and ∆K5ΔNβ-cateninER (ΔK5) (C, F) transgenic mice following treatment with 4OHT for 21 days. Scale bars: 50 µm.

The different phenotypes of K15ΔNβ-cateninER and ΔK5ΔNβ-cateninER transgenic mice did not reflect differences in the proliferative response to β-catenin (Fig. 6). Ki67 labeling revealed extensive proliferation at the base of anagen follicles in both models (Figs. 6B, C, E, F). In K15ΔNβ-cateninER transgenics there was extensive proliferation of bulge cells (Figs. 6B, E), while in ΔK5ΔNβ-cateninER transgenics there was proliferation associated with areas of developing ectopic follicles (Figs. 6C, F).

### Expression of markers of HF, SG and IFE

**Fig. 7:**
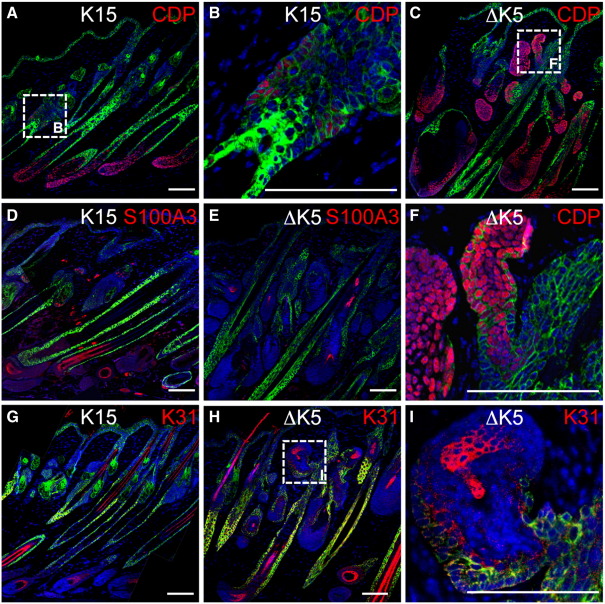
Expression of hair follicle differentiation markers. Immunofluorescence staining of K15ΔNβ-cateninER (K15) and ∆K5ΔNβ-cateninER (ΔK5) transgenic back skin following 21 days of 4OHT treatment. Sections were labeled with antibodies to (A–C, F) CDP (red), (D, E) S100A3 (red) or (G–I) K31 (red), and keratin 14 (green), with DAPI nuclear counterstain (blue). Scale bars: 50 µm.

**Fig. 8:**
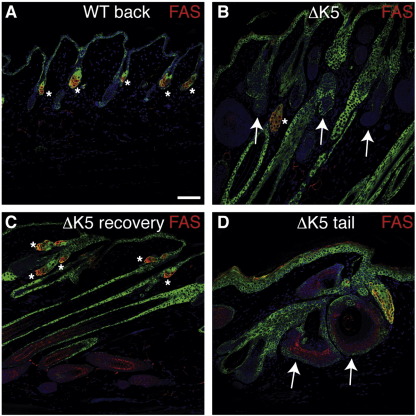
Sebaceous gland differentiation. Immunofluorescence staining of wild type (WT) and ∆K5ΔNβ-cateninER (ΔK5) transgenic back (A–C) and tail (D) skin following 21 days of 4OHT treatment. Skin in (C) was left untreated for a further 21 days. Sections were labeled with antibodies fatty acid synthase (red) and keratin 14 (green), with DAPI nuclear counterstain (blue). Asterisks indicate fatty acid synthase positive regions. Arrows in (B) indicate ectopic HF arising from SG. Arrows in (D) indicate cysts. Scale bar: 50 µm.

To characterize the changes induced by β-catenin activation, we examined expression of a range of lineage markers. Keratin 10, filaggrin and loricrin were used as markers of IFE (data not shown; Niemann et al., 2002). CCAAT displacement protein (CDP) is expressed in early ectopic follicles and in the inner root sheath and companion layer of mature follicles (Figs. 7A–C, F; Silva-Vargas et al., 2005). S100A3 (Figs. 7D, E) and K31 (hHa1) (Figs. 7G–I) were examined as markers of the cuticle and cortical layers of the hair follicle, respectively (Pálmer et al., 2008a). Fatty acid synthase and the lipophilic dye Nile Red were examined as markers of mature sebocytes (Fig. 8 and data not shown; Lo Celso et al., 2004; Berta et al., 2009).

The only lineage marker to be expressed in the enlarged bulge of K15ΔNβ-cateninER follicles was CDP (Figs. 7A, B, D, G and data not shown). CDP was widely expressed in ectopic follicles in ΔK5ΔNβ-cateninER skin (Figs. 7C, F), while K31 and S100A3 were expressed in a subset of ectopic follicles (Figs. 7E, H, I). Ectopic ΔK5ΔNβ-cateninER follicles did not express K10 (data not shown).

Fatty acid synthase was highly expressed in all sebaceous glands of wild type and untreated transgenic epidermis (Fig. 8A and data not shown). In contrast, expression of fatty acid synthase was almost undetectable by 14 days of 4OHT treatment in ΔK5ΔNβ-cateninER mice, correlating with the conversion of essentially all SG into hair follicles (Fig. 8B, arrows; Fig. 5J). The few areas of fatty acid synthase expression that remained were in residual SG (Fig. 8B, asterisk). When mice were treated with 4OHT for 21 days and examined 21 days later, ectopic follicles had largely regressed (Fig. 5L); SGs had reformed and were fatty acid synthase positive (Fig. 8C, asterisks). The cysts that formed in tail epidermis of ΔK5ΔNβ-cateninER mice labeled positive for Nile Red, fatty acid synthase, CDP and occasionally K31, but not for K10 (Fig. 8D and data not shown) and thus exhibited elements of both sebocyte and HF differentiation.

We conclude that β-catenin activation does not induce expression of markers of mature SG, IFE or HF lineages in the bulge. β-catenin activation in the SG results in a switch from SG differentiation to HF differentiation. In the tail a subset of SG retain their differentiated characteristics, while also expressing HF markers and these have the morphological appearance of cysts. This contrasts with the mixed IFE and SG lineages in cysts that form on overexpression of N-terminally truncated dominant negative Lef1 (Niemann et al., 2002).

### Expression of bulge and junctional zone stem cell markers

**Fig. 9:**
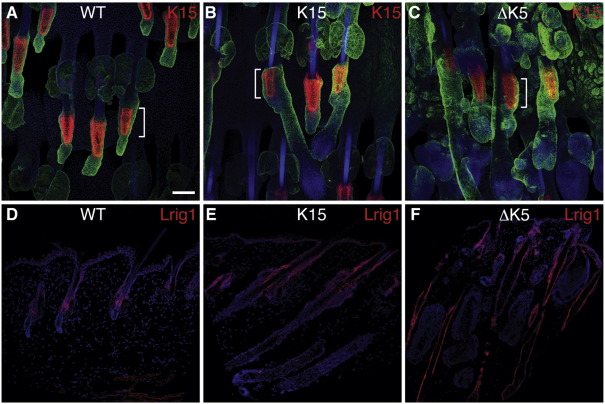
Expression of stem cell markers K15 and Lrig1. Immunofluorescence staining of wild type (WT), K15ΔNβ-cateninER (K15) and ∆K5ΔNβ-cateninER (ΔK5) transgenic skin following 21 days of 4OHT treatment. (A–C) Tail skin whole mounts labeled for K15 (red) and K14 (green) with DAPI nuclear counterstain (blue). Brackets indicate position of bulge in existing follicles. (D–F) Back skin sections labeled with anti-Lrig1 (red) with DAPI nuclear counterstain (blue). Scale bar: 50 µm.

Multiple pools of stem cells have now been identified in different regions of the epidermis, including the bulge of the hair follicle and the junctional zone that lies between the hair follicles, sebaceous glands and infundibulum of the interfollicular epidermis (reviewed by Watt and Jensen, 2009). It was previously reported that β-catenin-induced ectopic hair follicle formation does not affect expression of the bulge marker K15 in existing hair follicles (Silva-Vargas et al., 2005). In contrast, β-catenin activation increases the size of the junctional zone stem cell compartment, and loss of Lrig1, which is normally highly expressed in the junctional zone, causes a selective increase in β-catenin-induced ectopic hair follicle formation in the interfollicular epidermis (Jensen et al., 2009). These observations led us to investigate whether selective β-catenin activation in the bulge or SG and hair follicle bulb affected expression of K15 and Lrig1 (Fig. 9).

We examined K15 expression in whole mounts of tail epidermis (Braun et al., 2003) and in conventional sections of back skin (Figs. 9A–C and data not shown). 4OHT treatment of K15ΔNβ-cateninER or ΔK5ΔNβ-cateninER back or tail skin did not cause any change in K15 expression in the hair follicle bulge (Figs. 9A–C and data not shown). In contrast, 4OHT treatment of K15ΔNβ-cateninER skin resulted in increased Lrig1 expression in the infundibulum (Figs. 9D, E). 4OHT treatment of ΔK5ΔNβ-cateninER back skin also led to increased Lrig1 expression; however, in this case expression not only extended upwards into the infundibulum, but also downwards along the ORS of anagen follicles and into ectopic follicles (Fig. 9F). Thus, β-catenin activation does not affect expression of K15 within existing follicles but results in upregulation of Lrig1, regardless of which promoter is used to drive transgene expression.

### Vitamin D treatment stimulates differentiation of the HF lineages

It has previously been reported that one third of epidermal β-catenin target genes lack Lef/Tcf sites but contain vitamin D response elements, and are regulated by direct interactions between β-catenin and the vitamin D receptor (Cianferotti et al., 2007; Pálmer et al., 2008a). Combined treatment of K14ΔNβ-cateninER mice with 4OHT and an analogue of the active form of vitamin D (1,25-dihydroxyvitamin D3) promotes ectopic hair follicle differentiation to a greater extent than treatment with 4OHT alone (Pálmer et al., 2008a). We therefore examined whether treatment with a 1,25-dihydroxyvitamin D3 analogue (CD578; Wu et al., 2002) would alter the phenotype of K15ΔNβ-cateninER and ΔK5ΔNβ-cateninER mice.

**Fig. 10:**
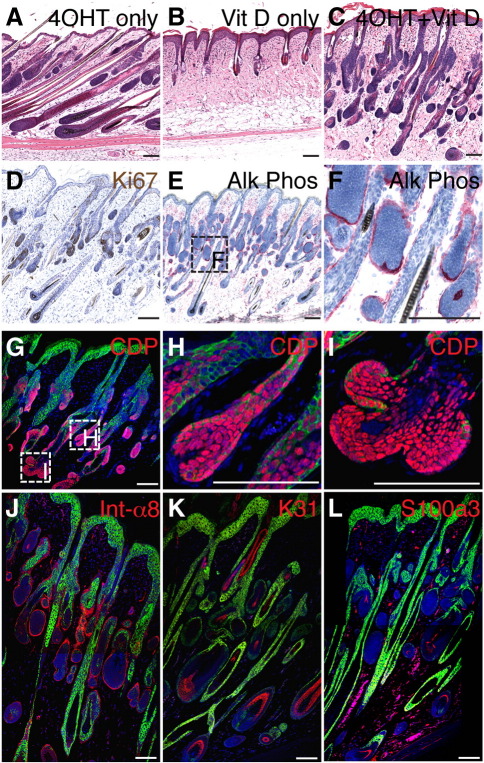
Effects of combined activation of β-catenin and vitamin D receptor. Back skin of ∆K5ΔNβ-cateninER transgenic mice was treated with (A) 4OHT alone, (B) 1,25-dihydroxyvitamin D3 analogue alone (Vit D) or (C–L) 4OHT and 1,25-dihydroxyvitamin D3 analogue for 14 days. (A–C) H&E stained sections. (D) Ki67 immunohistochemical staining. (E, F) Histochemical detection of alkaline phosphatase activity (red). (G–L) Immunofluorescence staining of (G–I) CDP (red), (J) (α8 integrin (red), (K) K31 (red), (L) S100A3 (red) and keratin 14 (green), with DAPI nuclear counterstain (blue). Scale bars: 50 µm.

CD578 treatment of wild type and transgenic skin led to a thickening of the IFE but did not affect the SG and HF (Fig. 10B and data not shown). Combined treatment of K15ΔNβ-cateninER mice with 4OHT and the 1,25-dihydroxyvitamin D3 analogue led to a reduction in Ki67 labeling in the bulge, but otherwise had no discernible effect compared to 4OHT treatment alone (data not shown). In contrast, ΔK5ΔNβ-cateninER mice showed a profoundly different phenotype when treated with both 4OHT and CD578 (Fig. 10C) as opposed to 4OHT alone (Fig. 10A). In the presence of the 1,25-dihydroxyvitamin D3 analogue proliferation of ectopic follicles was decreased (Fig. 10D; compare Fig. 6C) and the follicles arising from the SG formed mature, alkaline phosphatase-positive dermal papillae that were encircled by the adjacent epithelium (Figs. 10E, F, H, J; Lo Celso et al., 2004; Handjiski et al., 1994). HF differentiation was stimulated, as judged by increased expression of K31 (Fig. 10K), S100A3 (Fig. 10L), and the appearance of mature hair shafts (Fig. 10C). In addition, 1,25-dihydroxyvitamin D3 treatment led to an increase in the number of ectopic follicles arising out of the ORS and bulb (Table 1); this is illustrated in Fig. 10I, which shows a hair bulb with two ectopic CDP positive outgrowths. We conclude that concomitant activation of β-catenin and the vitamin D receptor promotes hair follicle differentiation at the expense of proliferation.

## Discussion

In order to compare the sensitivity of different adult epidermal subpopulations to β-catenin activation, we targeted a 4-hydroxy-tamoxifen (4OHT) inducible, stabilised β-catenin transgene to the hair follicle bulge (K15ΔNβ-cateninER) or to the outer root sheath and sebaceous glands (ΔK5ΔNβ-cateninER). Activation of either transgene triggered anagen entry, but had no effect on IFE proliferation and differentiation. The lack of ectopic follicles in the interfollicular epidermis is consistent with our earlier observation (Silva-Vargas et al., 2005) that ectopic follicles induced in the IFE by β-catenin activation are derived from IFE cells, not from migration of cells from the HF, as subsequently confirmed by others (Ito et al., 2007).

While it is well established that Wnt signalling is required to initiate anagen (DasGupta and Fuchs, 1999; Van Mater et al., 2003; Lo Celso et al., 2004; Lowry et al., 2005), our experiments are the first to show that activation of β-catenin exclusively in the bulge is sufficient to drive anagen. It is more surprising that activation of β-catenin in cells expressing the ΔK5 promoter triggered anagen, since the ΔK5 promoter was not active in the bulge: no ΔK5-driven transgene expression was detected by in situ hybridisation or antibody staining; transgene activation did not stimulate bulge cells to proliferate; and there was no induction of CDP, Lef1 or Jagged1 in the bulge of ΔK5ΔNβ-cateninER follicles.

There are a number of possible explanations for how non-bulge hair follicle cells could drive anagen. First, β-catenin activation could induce conversion into hair follicle stem cells; one precedent for that is β-catenin mediated induction of IFE cells to express bulge stem cell markers such as CD34 (Silva-Vargas et al., 2005). The β-catenin responsive cells would not necessarily be stem cells themselves, given that committed IFE progenitors can exhibit multi-lineage differentiation potential in epidermal reconstitution assays (Jensen et al., 2009). Secondly, the cells driving anagen could be a distinct, pre-existing stem cell population; in support of this, a growing number of stem cell populations outside the bulge have now been described (Watt and Jensen, 2009; Snippert et al., 2010) and there has long been evidence for the existence of a distinct population of stem cells in the germinative compartment of the secondary hair germ (Panteleyev et al., 2001). Finally, cells are known to move downwards from the bulge during normal anagen (Braun et al., 2003; Tumbar et al., 2004; Jaks et al., 2008) and β-catenin mediated stimulation of proliferation and downgrowth of the follicle bulb could stimulate replenishment by hair follicle bulge cells (Greco et al., 2009).

The most striking difference between 4OHT treated ΔK5ΔNβ-cateninER and K15ΔNβ-cateninER skin was that only the ΔK5 promoter triggered ectopic follicle formation. Activation of β-catenin in the bulge failed to induce ectopic follicles, even though Lef1 and Jagged1 were upregulated and proliferation was stimulated, and expression of the bulge marker K15, which is expressed both by quiescent and actively cycling (Lgr5-positive) bulge stem cells (Watt and Jensen, 2009), was unaffected. Our findings fit well with the observation that ablation of expression of BMP receptor 1a causes expansion of the bulge, proliferation and upregulation of Lef1 and β-catenin signalling, yet does not induce ectopic follicles (Zhang et al., 2006; Kobielak et al., 2007).

The bulge differs from the rest of the epidermis in expressing Tcf3 and 4, which are required for hair follicle maintenance (Merrill et al., 2001; Nguyen et al., 2009) and therefore the set of genes activated by ß-catenin in the bulge will differ from those in the SG and ORS. Expression of the Notch ligand Jagged1 is required for ectopic follicle formation (Estrach et al., 2006) and Jagged1 was only weakly upregulated in the bulge of K15ΔNβ-cateninER hair follicles. However, this cannot account for the lack of ectopic follicles, because when we examined the epidermis of mice in which both β-catenin and Notch were activated we only found very occasional outgrowths from the bulge (Estrach et al., 2006; Baker and Watt, unpublished).

Another likely explanation for the lack of ectopic follicles originating in the bulge is that the local microenvironment is not permissive for differentiation. The bulge microenvironment has several unique characteristics, both in terms of expression of adhesion molecules and in terms of the proximity of non-epidermal cells, such as melanocyte stem cells and immature Langerhans cells (Cotsarelis, 2006). The adhesive properties of the bulge may ensure that cells exiting the bulge move downwards, except following wounding or during tumour initiation (Ito et al., 2005, 2007; Malanchi et al., 2008). For example, the intercellular adhesion protein Nectin-like molecule 2 (Necl2) contributes to bulge cell adhesion and not only promotes stem cell quiescence but also restricts cell motility (Giangreco et al., 2009). The progeny of K15ΔNβ-cateninER positive cells that exit the bulge no longer express the transgene and so are unable to form ectopic follicles in the ORS or bulb.

In contrast to the bulge, the base of the SG is a highly permissive environment for ectopic follicle formation and follicles were induced in that location after a single dose of 4OHT. 4OHT treatment of ΔK5ΔNβ-cateninER skin led to proliferation of cells in the SG. As ectopic follicle formation proceeded, all differentiated sebocytes were lost from the SG; however, on withdrawal of 4OHT differentiation of sebocytes resumed. Two markers of SG stem cells have been described: the EGFR antagonist Lrig1 and the transcriptional co-repressor Blimp1. Blimp1 is not reported to be expressed in the region where new follicles originated (Horsley et al., 2006). However, Lrig1 is expressed by multi-potent, Lgr6-positive stem cells in the junctional zone (Jensen et al., 2009; Snippert et al., 2010) and these cells are known to expand on β-catenin activation (Jensen et al., 2009). 4OHT treatment of ΔK5ΔNβ-cateninER transgenics resulted in increased Lrig1 expression (Fig. 8F), which could either reflect expansion of the stem cell compartment or Myc-dependent upregulation as a result of increased proliferation (Fig. 6C; Jensen et al., 2009). Further analysis is required to determine whether the β-catenin responsive cells at the base of the SG are a new stem cell population or whether there are unique characteristics of their location that are permissive to ectopic follicle formation.

Treatment with a 1,25-dihydroxyvitamin D3 analogue did not over-ride the block in ectopic follicle formation of bulge stem cells, but enhanced differentiation of SG-derived hair follicles. This is consistent with the observation that epidermal cells that lack the VDR exhibit increased sebocyte differentiation (Cianferotti et al., 2007; Luderer and Demay, 2010). The 1,25-dihydroxyvitamin D3 analogue stimulated ectopic follicle formation in the ORS below the bulge and also in the HF bulb, which is in good agreement with the observation that 1,25-dihydroxyvitamin D3 enhances HF differentiation when active β-catenin is expressed throughout the basal layer of the epidermis (Pálmer et al., 2008a). The recent finding that CDP is a ligand-dependent co-activator of the VDR (Ochiai et al., 2009) suggests that CDP may play a positive role in hair follicle formation that has not been appreciated previously. One effect of 1,25-dihydroxyvitamin D3 may be to promote downward growth of ectopic follicles, since VDR null keratinocytes exhibit defective motility (Pálmer et al., 2008b).

One final issue is the relationship between β-catenin activation and tumour formation. Sustained β-catenin activity drives the formation of benign HF tumours called pilomatricomas in mouse models (Gat et al., 1998; Lo Celso et al., 2004) and the equivalent human tumours have activating β-catenin mutations (Chan et al., 1999). Lineage tracing shows that cells from the bulge can contribute to squamous cell carcinomas, and ablation of β-catenin via the K14 promoter results in regression of chemically induced papillomas (Malanchi et al., 2008). The lack of tumours in the K15ΔNβ-cateninER mice indicates that activation of β-catenin exclusively in the bulge is not sufficient to cause pilomatricomas, whether in undamaged tissue (Fig. 5) or following wounding (data not shown). Prolonged activation of β-catenin under the control of the ΔK5 promoter led to massive growth of hair follicle cells, but the phenotype was not as severe as in K14ΔNβ-cateninER mice (Lo Celso et al., 2004). This may indicate a contribution of IFE or junctional zone cells to tumour formation.

Taken together with previous observations (Silva-Vargas et al., 2005; Jensen et al., 2009), our results indicate that the SG, junctional zone and interfollicular epidermis are competent to form ectopic hair follicles in response to β-catenin activation, whereas the bulge is not. Responsiveness to Wnt signalling therefore depends not only on signal strength and duration (Lo Celso et al., 2004; Lowry et al., 2005; Silva-Vargas et al., 2005) but also on the status or location of the responding cells.

## Figures and Tables

**Table 1:** Quantitation of ectopic hair follicle formation in ΔK5ΔNβ-cateninER epidermis treated with 4OHT alone or in combination with vitamin D analogue (Vit D). EF: ectopic follicles; SG: sebaceous gland; ORS: outer root sheath; HB: hair bulb.

Condition	No. hair follicles scored	Total EF	EF from SG	EF in ORS	EF from HB
4OHT only	100	148	141 (95%)	7 (5%)	0 (0%)
4OHT + Vit D	95	261	138 (53%)	115 (44%)	8 (3%)
